# Strike of German Doctors

**Published:** 1914-07-11

**Authors:** 


### Strike of German Doctors.

It seems that the *Leipzige Verband's* stand last year on behalf of the German doctors against the *Krankenkassen* (approved societies) has not led to permanent peace between the contending parties. On July 6 the National Insurance doctors of a district near Berlin struck work as a protest against alleged attempted encroachment by the *Krankenkassen* upon their rights. More than a hundred doctors are affected directly, and a great many more indirectly; though it is not quite clear from the telegrams exactly what their grievances are. Still the incident is important as a symptom that antipathies still simmer, ready to boil over into open hostilities at any moment. The *Krankenkassen* appear to have been biding their time for a favourable opportunity of attacking the profession; and to have been refusing to make definite contracts, preferring the renewal from time to time of short-dated agreements. Obviously, if this is the case, it would be foolish for the doctors to wait to be attacked; to wait, that is, until some circumstance should put them in a position of disadvantage. In such circumstances it was sound strategy to assume the offensive at once. The lesson will, perhaps, not be wasted on the medical profession in this country.

